# Taxonomic diversity and abundance of enchytraeids (Annelida, Clitellata, Enchytraeida) in the Northern Palaearctic. 1. Asian part

**DOI:** 10.3897/BDJ.12.e114682

**Published:** 2024-01-03

**Authors:** Maxim I. Degtyarev, Ruslan A. Saifutdinov, Daniil I. Korobushkin, Alexander I. Bastrakov, Margarita A. Danilova, Ivan D. Davydov, Anastasia Yu. Gorbunova, Polina A. Guseva, Evgeniy I. Karlik, Roza E. Koshchanova, Ksenia G. Kuznetsova, Iurii M. Lebedev, Dmitriy A. Medvedev, Roman R. Obolenskiy, Anna V. Popova, Nina A. Pronina, Leonid B. Rybalov, Alexei V. Surov, Akmal B. Tadzhimov, Alexander I. Tarasov, Vladislav A. Vasiliev, Andrey S. Zaitsev, Elena Yu. Zvychaynaya, Konstantin B. Gongalsky

**Affiliations:** 1 A.N. Severtsov Institute of Ecology and Evolution RAS, Moscow, Russia A.N. Severtsov Institute of Ecology and Evolution RAS Moscow Russia; 2 Karakalpak State University named after Berdakh, Nukus, Uzbekistan Karakalpak State University named after Berdakh Nukus Uzbekistan; 3 University of Bergen, Bergen, Norway University of Bergen Bergen Norway; 4 Lomonosov Moscow State University, Moscow, Russia Lomonosov Moscow State University Moscow Russia

**Keywords:** sampling event, soil fauna, potworm, tundra, boreal, nemoral, steppe, desert, Siberia, Russian Far East, Uzbekistan, Kazakhstan, Mongolia, mesofauna, Enchytraeidae, soil fauna, terrestrial oligochaetes

## Abstract

**Background:**

Enchytraeids, or potworms, are tiny oligochaetes that are distributed worldwide in many terrestrial, freshwater and marine ecosystems. Despite their key role in the functioning of ecosystems, the diversity and abundance of Enchytraeidae are rarely studied due to the laborious process of species identification. The present study addresses this gap and sheds some light on the distribution and abundance of enchytraeids in the lands of the Northern Palearctic. The provided dataset constitutes the latest and comprehensive field sampling of enchytraeid assemblages across the Asiatic part of the Northern Palearctic, encompassing an original set of soil samples systematically collected throughout the region from 2019 to 2022.

**New information:**

The dataset includes occurrences from 131 georeferenced sites, encompassing 39 species and 7,074 records. This represents the first dataset providing species-specific information about the distribution and abundance of terrestrial enchytraeids across an extensive geographic area covering the Asian sector of the Northern Palaearctic. The compiled dataset is the key for exploring and understanding local and regional enchytraeid diversity. It may also serve as a valuable resource for monitoring and conserving the entire soil biodiversity.

## Introduction

Enchytraeids, also known as potworms, are tiny, yet ecologically impactful components of biota living in soils and freshwater and marine sediments worldwide (Erséus et al. 2010, Rota and de Jong 2015). Despite their small size and especially where earthworms are scarce or absent, they play a vital role in terrestrial ecosystems by regulating many key processes like nutrient cycling and maintaining soil structure (Potapov et al. 2022). However, due to the highly laborious taxonomic identification that involves in vivo morphological evaluation, as well as the considerable lack of experienced staff worldwide, there is a dramatic shortage of studies devoted to understanding their temporal and spatial distribution at the species level (Römbke et al. 2017). This is especially true for the eastern part of the Northern Palearctic, which remains as one of the least studied areas in terms of enchytraeids (Nurminen 1982, Rota and de Jong 2015). Nevertheless, the situation is changing and there is a growing interest in the ecology and taxonomy of Enchytraeidae in this part of the world (Rota et al. 2018, John et al. 2019, Degtyarev et al. 2020). The objective of this data paper is to address this knowledge gap. To achieve this, we conducted a field survey of enchytraeid fauna and population across various biomes within the Northern Palearctic between 2019 and 2022. Due to the extensive geographical extent and the significant amount of material that still requires identification, we have chosen to split the resulting dataset into two main parts: Asian and European. The dataset dedicated to the European part will be submitted to the same journal in the near future (Saifutdinov et al., in prep.).

## General description

### Purpose

The purpose of the data paper is to depict the distribution and abundance of enchytraeids in the Northern Palearctic Region, particularly in its Asiatic part.

## Project description

### Study area description

The area under investigation is the Asian part of the Northern Palearctic, encompassing a diverse range of biome types, starting from Siberian tundra in the far north to temperate forests and deserts in the south (Binney et al. 2017). We limit the research area to the Ural Mountains in the west, Uzbekistan and Mongolia in the south. The territory of China was excluded due to organisational reasons. In total, we examined 131 sites located within various biomes as classified by the WWF (Olson et al. 2001), including: (1) tundra, (2) boreal forests, (3) temperate coniferous forests, (4) temperate broadleaf and mixed forests, (5) temperate grasslands, savannahs and shrublands, (6) flooded grasslands and savannahs and (7) deserts and xeric shrublands. In each of the biomes, we collected from a different number of sites due to logistical constraints and various extraction capacities. Comprehensive information about each site is given in Table 1.

## Sampling methods

### Sampling description

The material for the dataset was collected between 2019 and 2022. We selected sampling sites in areas that were not heavily disturbed by human activity. In arid regions, we chose the most humid (but not flooded) spots. The sampling protocol was developed in compliance with widely recognised methods (Ghilarov 1975, Coleman et al. 2004). At each site, we collected a random selection of 1 to 7 soil monoliths. Detailed information on number of soil monoliths collected from each site can be found in the "samplingEffort" column within the GBIF dataset (Degtyarev et al. 2023). These soil monoliths were taken using a 5-cm-diameter steel corer down to a depth of 10 cm. After collection, the soil was carefully placed into plastic bags and transported to the laboratory at the A. N. Severtsov Institute of Ecology and Evolution, Russian Academy of Sciences, Moscow. Subsequently, the soil samples were stored in a refrigerator at +4°C until extraction. Enchytraeids were extracted from the soil using the wet funnel technique as described by Didden et al. (1995). It is commonly known as Graefe's method and is a somewhat simplified modification of O'Connor's method (O'Connor 1962). Graefe's innovation is the rejection of artificial heating of the surface of the soil sample. Otherwise, it is not significantly different in efficiency from the O'Connor method (Kobetičová and Schlaghamerský 2003). We placed a sieve in each funnel and a soil monolith in each sieve. Then tap water was poured into the funnel so that the soil monolith was completely covered. A test tube was attached to each funnel and placed in a container with room temperature water. This precaution aimed to prevent potential overheating of the extracted enchytraeids, considering the possibility of random and sudden temperature fluctuations in the extraction room. Extraction was carried out for 16 to 24 hours, after which the tubes were detached from the funnels and the contents of the tubes were poured into Petri dishes.

### Quality control

The samples were collected by a number of soil zoologists and ecologists from the A.N. Severtsov Institute of Ecology and Evolution, Russian Academy of Sciences, Moscow and trained volunteers. In total, 39 different enchytraeid species were collected. Given the variance in the number of soil monoliths across sites, the dataset includes abundance expressed as individuals per square metre. Enchytraeid species were identified in vivo immediately after the extraction procedure, according to Schmelz and Collado (2010). For species not included in this guide or described later, comparisons with original descriptions were used. We also employed molecular analysis to verify the species *Fridericiabulboides* and *Mesenchytraeusgigachaetus*. Detailed information on the methods used for molecular analysis is available in Degtyarev et al. (2020).

Some of the species we have found exhibit distinct morphological differences from all known enchytraeid species. We are confident that these species have not yet been described in literature. A comprehensive description of these species will be possible once more data have been collected. Therefore, we have decided to refer to them as *Fridericia* sp. 1, *Enchytraeus* sp. 1, *Henlea* sp. 1 and *Henlea* sp. 2 for now. *Henlea* sp. 1 and *Henlea* sp. 2 are large *Henlea* worms, both with unusually robust spermathecae. *Fridericia* sp. 1 is a medium-sized *Fridericia* species from mountainous Uzbekistan. *Enchytraeus* sp. 1 is possibly an obligate parthenogenetic species from the *E.buchholzi* group, characterised by underdeveloped male copulatory organs.

The taxonomy follows the WoRMS database (Timm and Erséus 2023). Scientific names were checked using the GBIF species matching tool. Subsequently, the identified enchytraeids were used for further molecular analyses (COI and/or H3 genes). As such, all instances of enchytraeid occurrences within the studied sites were recorded as dwc:basisOfRecord = "HumanObservation". Juvenile specimens were identified at the genus level. The identification of all enchytraeids was conducted by Maxim Degtyarev.

### Step description

1) The selection of study sites was driven by the intention to locate undisturbed areas displaying minimal or no signs of human activity.

2) Site sampling was carried out at a distance of no less than 100 m from the borders of selected zonal sites within one of the seven biome types according to WWF (Olson et al. 2001): tundra, boreal forests, temperate coniferous forests, temperate broadleaf and mixed forests, temperate grasslands, savannahs and shrublands, flooded grasslands and savannahs, as well as desert and xeric shrublands.

3) At each site, soil monoliths were collected using a steel corer with a diameter of 5 cm, reaching a depth of 10 cm.

4) The transportation of soil monoliths was conducted in isothermic containers to prevent soil overheating, which could lead to the mortality of organisms present.

5) Enchytraeids were extracted from the soil using the wet funnel method as described by Didden et al. (1995).

6) Following the extraction process, enchytraeids were identified in vivo to the species level using an Olympus BX-43 microscope. Subsequently, they were preserved in 96% alcohol for further molecular and isotopic analyses.

## Geographic coverage

### Description

**Figure 1.**
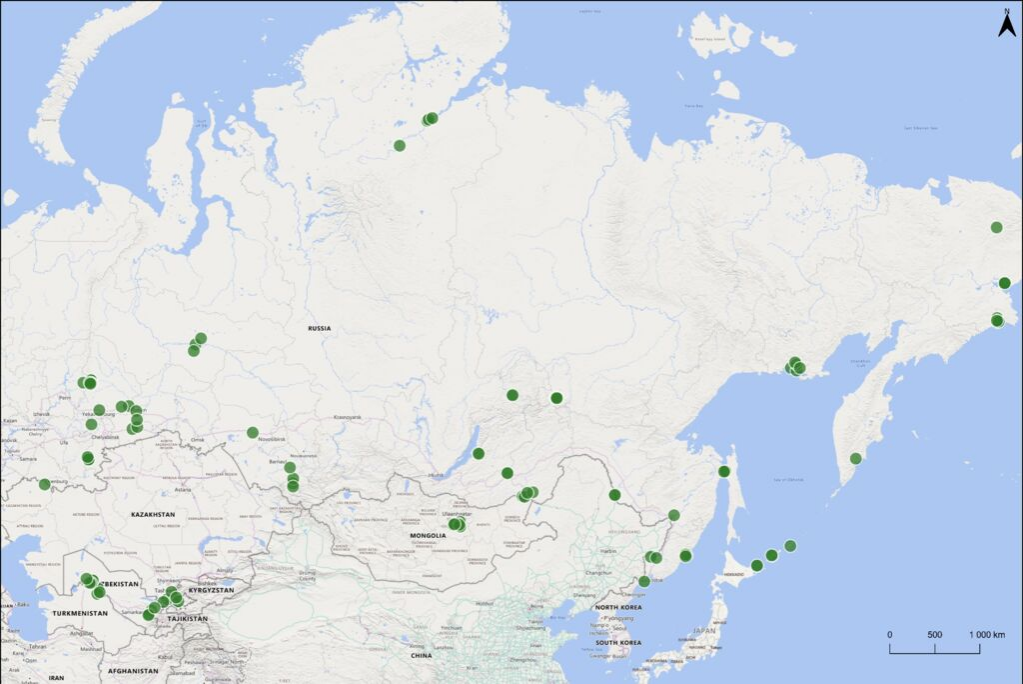
Enchytraeidae sampling locations in the Asian part of the Northern Palaearctic. The map was created using QGIS 3.32.2 - Lima software (QGIS.org 2023).

The research region was located in the Asian part of the Northern Palearctic, from the Ural Mountains in the west to the Pacific coast in the Russian Far East (Fig. 1). It included biomes in West and East Siberian Russia, Kazakhstan, Mongolia, Uzbekistan and the Russian Far East. This extensive geographic area consists of diverse habitat types, including tundra, taiga, steppe, boreal forest and mountain ranges. Spanning a large latitudinal gradient, the region contains hot desert (BWh), cold desert (BWk), hot semi-arid (BSh) and cold semi-arid (BSk) climates in the south, transitioning to humid continental (Dfa) and warm summer continental (Dfb) climates in the mid-latitudes and subarctic (Dfc) and tundra (ET) climates in the far north near the Arctic, according to the Köppen-Geiger climate classification (Beck et al. 2018).

The geographical references were obtained by recording the coordinates of the sampling sites using a mobile phone and the Organic Maps app (Organic Maps OÜ 2023). The measurement error of the coordinates was approximately 25 m. The WGS84 coordinate system was used for all records.

### Coordinates

39.3147 and 72.4874 Latitude; 53.5298 and 177.8474 Longitude.

## Taxonomic coverage

### Description

Across the 131 sites studied within seven biomes in the Asiatic part of the Northern Palaearctic, we identified a total of 39 species belonging to 16 genera. The highest species richness was recorded in boreal forests (34 species in total, see Table 2). Temperate broadleaf and mixed forests, as well as grasslands and shrublands, hosted approximately 20 species each. In the tundra biome, we found 16 species. The number of species in temperate coniferous forests, flooded grasslands and savannahs ranged from 10 to 11. The lowest species richness was observed in xeric shrublands and deserts (Table 2).

The average species richness of enchytraeids varied between four species per site in flooded grasslands and savannahs and 0.25 species per site in deserts and xeric shrublands. The same trends were also observed in the case of the average abundance of enchytraeids (see Table 2).

### Taxa included

**Table:** 

Rank	Scientific Name	Common Name
family	Enchytraeidae	pot worm

## Temporal coverage

**Data range:** 2019-7-11 – 2022-11-01.

## Usage licence

### Usage licence

Other

### IP rights notes

This work is licensed under a Creative Commons Attribution 4.0 International License (CC BY 4.0).

## Data resources

### Data package title

Taxonomic diversity and abundance of enchytraeids (Annelida: Clitellata: Enchytraeida) in the Northern Palaearctic. 1. Asian part

### Resource link

https://doi.org/10.15468/appcnv

### Alternative identifiers

https://www.gbif.org/dataset/918d7d69-1626-4980-9f6a-74d04da30fde; http://gbif.ru:8080/ipt/resource?r=enchytraeids_2

### Number of data sets

1

### Data set 1.

#### Data set name

Taxonomic diversity and abundance of enchytraeids (Annelida: Clitellata: Enchytraeida) in the Northern Palaearctic. 1. Asian part

#### Data format

Darwin Core Archive format (https://ipt.gbif.org/manual/en/ipt/latest/dwca-guide)

#### Download URL

http://gbif.ru:8080/ipt/archive.do?r=enchytraeids_2&v=1.7

#### Description

The dataset includes two related tables linked by the eventID column - Sampling Events and Associated Occurrences. The Sampling Events table consists of 131 events. The Associated Occurrences table consists of 7,074 occurrences (Degtyarev et al. 2023).

**Data set 1.** 

Column label	Column description
eventID (Event core, Occurrence extension)	Each event is assigned a unique identifier constructed from the sampling date, country code, region abbreviation for Russia or full name for other countries and sampling site number. For example, the identifier "03-05-2022-UZ-Karakalpakstan-70" corresponds to the event recorded on 3 May 2022, at sampling site #70 in Karakalpakstan, Uzbekistan.
eventDate (Event core)	Date on which soil samples were collected, formatted as YYYY-MM-DD (year-month-day) according to ISO 8601.
day (Event core)	The integer day of the month on which the dwc:Event occurred.
month (Event core)	The integer month in which the dwc:Event occurred.
year (Event core)	The four-digit year in which the dwc:Event occurred, according to the Common Era Calendar.
samplingProtocol (Event core)	A constant value describing the extraction method used for all sampling events. The protocol was wet extraction of animals from 19.6 cm^2^ soil cores using funnels.
samplingEffort (Event core)	The number of soil samples collected and processed using the extraction procedure.
sampleSizeValue (Event core)	A numeric value for a measurement of the size (volume) of a sample in a sampling dwc:Event.
sampleSizeUnit (Event core)	The unit of measurement of the size (volume) of a sample in a sampling dwc:Event.
locality (Event core)	The name of the closest town, village or other significant human settlement near the sampling site.
decimalLatitude (Event core)	The geographic latitude (in decimal degrees, using the spatial reference system given in dwc:geodeticDatum) of the geographic centre of a dcterms:Location.
decimalLongitude (Event core)	The geographic longitude (in decimal degrees, using the spatial reference system given in dwc:geodeticDatum) of the geographic centre of a dcterms:Location.
coordinatePrecision (Event core)	A decimal representation of the precision of the coordinates given in the dwc:decimalLatitude and dwc:decimalLongitude.
coordinateUncertaintyInMeters (Event core)	The horizontal distance (in metres) from the given dwc:decimalLatitude and dwc:decimalLongitude describing the smallest circle containing the whole of the dcterms:Location.
geodeticDatum (Event core)	The ellipsoid, geodetic datum or spatial reference system (SRS), upon which the geographic coordinates given in dwc:decimalLatitude and dwc:decimalLongitude are based. Constant value - "WGS84".
country (Event core)	The name of the country or major administrative unit in which the dcterms:Location occurs.
countryCode (Event core)	The standard code for the country in which the dcterms:Location occurs.
stateProvince (Event core)	The name of the next smaller administrative region than country (state, province, canton, department, region etc.), in which the dcterms:Location occurs. For sampling events in Russia, this records the federal subject (republic, krai, oblast etc.) where the sample was collected. For sampling events in other countries, this records administrative regions according to Database of Global Administrative Areas.
habitat (Event core)	This variable provides the biome classification assigned to the sampling location, based on the habitat typing system defined by the World Wildlife Fund (WWF). For additional information about the WWF biome classification system, please refer to Olson et al. (2001).
type (Event core)	The nature or genre of the resource. Constant value - event.
occurenceID (Occurrence extension)	Each occurrence is assigned a unique identifier constructed from the sampling date, country code, region abbreviation for Russia or full name for other countries, sampling site number and occurrence number at that site. For example, the identifier "12-07-2019-RU-SL-5-14" corresponds to the 14^th^ occurrence recorded on 12 July 2019 at sampling site #5 in Sakhalin Oblast, Russia.
basisOfRecord (Occurrence extension)	This field contains a constant value indicating the record type. All occurrences have the value "Human observation" because organisms were identified in vivo and then used for further molecular and isotopic analyses after collection.
recordedBy (Occurrence extension)	The person, group or organisation responsible for originally recording the occurrence data. For example: "Korobushkin D | Saifutdinov R".
identifiedBy (Occurrence extension)	The person, group or organisation responsible for identification. For all records in this dataset, organisms were identified by Maxim Degtyarev.
organismQuantity (Occurrence extension)	A number or enumeration value for the quantity of dwc:Organisms.
organismQuantityType (Occurrence extension)	The type of quantification system used for the quantity of dwc:Organisms.
occurenceStatus (Occurrence extension)	A statement about the presence or absence of a dwc:Taxon at a dcterms:Location.
taxonRemarks (Occurrence extension)	Freeform remarks entered relevant to the taxonomy and characterisation of the documented species or taxon. Example: "*Henleacf.nasuta*".
scientificName (Occurrence extension)	The full scientific name, with authorship and date information, if known. Example: "*Henleanasuta* (Eisen, 1878)".
kingdom (Occurrence extension)	The full scientific name of the kingdom in which the dwc:Taxon is classified.
phylum (Occurrence extension)	The full scientific name of the phylum or division in which the dwc:Taxon is classified.
class (Occurrence extension)	The full scientific name of the class in which the dwc:Taxon is classified.
order (Occurrence extension)	The full scientific name of the order in which the dwc:Taxon is classified.
family (Occurrence extension)	The full scientific name of the family in which the dwc:Taxon is classified.
genus (Occurrence extension)	The full scientific name of the genus in which the dwc:Taxon is classified.
scientificNameAuthorship (Occurrence extension)	The authorship information for the dwc:scientificName formatted according to the conventions of the applicable dwc:nomenclaturalCode.
taxonRank (Occurrence extension)	The taxonomic rank of the most specific name in the dwc:scientificName.

## Figures and Tables

**Table 1.** Locations, habitat information and number of recorded enchytraeid species for sampling sites in the Asian Northern Palaearctic Region.

**Biome**	**Site ID**	**Vegetation**	**Species Recorded**
Tundra	12-07-2020-RU-CK-23	Flood plain meadow	4
Tundra	12-07-2020-RU-CK-24	*Alnus* krummholz	3
Tundra	15-07-2020-RU-KK-27	Tundra	3
Tundra	16-07-2020-RU-KK-28	Tundra	2
Tundra	13-07-2020-RU-KK-29	Tundra	1
Tundra	08-07-2021-RU-CK-31	*Alnus* krummholz	0
Tundra	08-07-2021-RU-CK-32	*Alnus* krummholz	1
Tundra	10-07-2021-RU-CK-33	*Alnus* krummholz	4
Tundra	08-07-2021-RU-CK-35	*Alnus* krummholz	4
Tundra	12-07-2021-RU-CK-61	*Sphagnum* - *Rubuschamaemorus* tundra	1
Tundra	09-07-2021-RU-CK-62	*Salix* thicket	4
Tundra	08-07-2021-RU-CK-63	*Salix* thicket	3
Tundra	10-05-2022-RU-KK-88	Low-growing *Pinus* shrubland	7
Boreal forest	11-07-2019-RU-SL-1	*Larix* forest with *Pinuspumilia*	2
Boreal forest	11-07-2019-RU-SL-2	Coniferous forest with *Pinuspumilia*	1
Boreal forest	12-07-2019-RU-SL-5	Taiga forest with *Larixgmelinii*	2
Boreal forest	28-07-2019-RU-MG-12	*Larix* forest	3
Boreal forest	09-08-2019-RU-SL-13	Coniferous shrubland with *Sasakurilensis*	9
Boreal forest	28-07-2019-RU-MG-14	*Alnus* forest with high grass	4
Boreal forest	26-07-2019-RU-MG-19	*Larix* forest	0
Boreal forest	27-07-2019-RU-MG-22	*Larix* forest	2
Boreal forest	27-07-2020-RU-MG-25	*Larix* forest	1
Boreal forest	23-08-2021-RU-YA-39	*Pinuspumilia* woodland	8
Boreal forest	23-08-2021-RU-YA-40	*Pinuspumilia* woodland	2
Boreal forest	23-08-2021-RU-YA-41	*Pinuspumilia* woodland	1
Boreal forest	21-08-2021-RU-YA-42	*Larix* - dwarf *Betula* woodland	2
Boreal forest	26-08-2021-RU-YA-43	*Pinuspumilia* woodland	3
Boreal forest	25-08-2021-RU-YA-44	*Larix* - *Pinuspumilia* woodland	2
Boreal forest	26-08-2021-RU-YA-45	*Pinuspumilia* woodland	3
Boreal forest	21-08-2021-RU-YA-46	*Larix* - dwarf *Betula* woodland	2
Boreal forest	26-08-2021-RU-YA-47	*Pinuspumilia* woodland	1
Boreal forest	23-08-2021-RU-YA-48	*Larix* - dwarf *Betula* woodland	1
Boreal forest	26-08-2021-RU-YA-49	*Pinuspumilia* woodland	1
Boreal forest	21-08-2021-RU-YA-50	*Larix* - dwarf *Betula* woodland	1
Boreal forest	23-08-2021-RU-YA-51	*Larix* - dwarf *Betula* woodland	1
Boreal forest	25-08-2021-RU-YA-52	*Larix* - *Pinus*pumilia woodland	0
Boreal forest	25-08-2021-RU-YA-53	*Larix* - *Pinus*pumilia woodland	2
Boreal forest	21-08-2021-RU-YA-54	*Larix* - dwarf *Betula* woodland	2
Boreal forest	21-08-2021-RU-YA-55	*Larix* - dwarf *Betula* woodland	2
Boreal forest	24-08-2021-RU-YA-56	*Larix* - *Pinuspumilia* woodland	2
Boreal forest	23-08-2021-RU-YA-57	*Larix* - dwarf *Betula* woodland	2
Boreal forest	24-08-2021-RU-YA-58	*Larix* - *Pinuspumilia* woodland	2
Boreal forest	21-08-2021-RU-YA-59	*Larix* - dwarf *Betula* woodland	1
Boreal forest	24-08-2021-RU-YA-60	*Larix* - *Pinuspumilia* woodland	0
Boreal forest	16-06-2021-RU-IR-64	*Pinus* forest	4
Boreal forest	16-06-2021-RU-IR-65	*Pinus* forest	4
Boreal forest	16-09-2021-RU-SV-66	*Urtica* thickets	1
Boreal forest	06-06-2022-RU-HM-79	*Pinus* forest with *Betula*	2
Boreal forest	06-06-2022-RU-HM-80	*Pinus* forest with *Betula*	2
Boreal forest	06-06-2022-RU-HM-81	*Pinus* forest	3
Boreal forest	21-06-2022-RU-SV-83	*Betula* - *Pinus* forest	4
Boreal forest	21-06-2022-RU-SV-85	*Betula* - *Pinus* - *Picea* forest	6
Boreal forest	09-05-2022-RU-KT-91	Coniferous forest	4
Boreal forest	27-09-2022-RU-SV-104	*Betula* - *Populus* forest belt with *Salix* and Mixed Grasses	4
Boreal forest	27-09-2022-RU-SV-105	*Populus* forest with Mixed Grasses	4
Boreal forest	27-09-2022-RU-SV-106	Scattered forest on Anthropogenic Soils	2
Boreal forest	27-09-2022-RU-SV-107	*Tilia* - *Betula* - *Abies* forest	3
Boreal forest	27-09-2022-RU-SV-108	*Betula* forest	4
Boreal forest	28-09-2022-RU-SV-109	*Betula* - *Pinus* forest	3
Boreal forest	28-09-2022-RU-SV-110	Mixed fen forest	5
Boreal forest	28-09-2022-RU-SV-111	Lowland Sedge Bog with *Salix*	1
Boreal forest	28-09-2022-RU-SV-112	Mixed Coniferous and Deciduous fen forest	2
Boreal forest	29-09-2022-RU-SV-113	*Populus* - *Betula* forest	0
Boreal forest	30-09-2022-RU-SV-114	*Picea* - *Pinus* - *Abies* forest	3
Boreal forest	30-09-2022-RU-SV-115	*Picea* forest	3
Boreal forest	30-09-2022-RU-SV-116	*Betula* - *Abies* - Spruce forest	4
Boreal forest	30-09-2022-RU-SV-117	*Betula* - *Abies* forest	2
Boreal forest	30-09-2022-RU-SV-118	*Betula* - *Abies* forest	2
Boreal forest	20-08-2022-RU-ZK-125	*Pinus* forest with *Populus*	3
Boreal forest	20-08-2022-RU-ZK-126	Meadow with *Salix*	10
Boreal forest	18-08-2022-RU-BU-130	*Pinus* forest	0
Boreal forest	18-08-2022-RU-BU-131	Steppe	0
Temp. conifer. forest	03-08-2021-RU-AL-36	*Betula* forest	3
Temp. conifer. forest	02-08-2021-RU-AL-37	*Pinus* forest	5
Temp. conifer. forest	01-08-2021-RU-AL-38	Siberian *Pinus* - *Picea* forest	1
Temp. conifer. forest	10-07-2022-RU-NS-89	Deciduous forest	4
Temp. conifer. forest	13-07-2022-RU-AL-90	Deciduous forest	8
Temp. broadleaf & mixed forest	19-08-2019-RU-PK-8	Sparse *Quercus* forest	7
Temp. broadleaf & mixed forest	13-08-2019-RU-PK-9	*Quercus* - *Betula* forest	8
Temp. broadleaf & mixed forest	14-08-2019-RU-PK-11	Sparse *Betula* forest	7
Temp. broadleaf & mixed forest	14-08-2019-RU-PK-18	*Quercus* - *Betula* forest	5
Temp. broadleaf & mixed forest	20-08-2019-RU-SL-20	*Abies* and *Picea* forest	7
Temp. broadleaf & mixed forest	21-08-2019-RU-SL-21	*Sasakurilensis* thicket	1
Temp. broadleaf & mixed forest	12-06-2020-RU-SL-26	Broadleaf forest	4
Temp. broadleaf & mixed forest	12-06-2020-RU-SL-30	*Picea* - *Abies* forest	2
Temp. broadleaf & mixed forest	18-06-2022-RU-KN-82	*Pinus* forest with *Betula* and *Vacciniumvitis-idaea*	0
Temp. broadleaf & mixed forest	19-06-2022-RU-TN-84	*Pinus* forest	1
Temp. broadleaf & mixed forest	18-06-2022-RU-KN-87	*Betula* - *Pinus* forest	2
Temp. grasslands & savannas	27-07-2019-RU-ZK-3	Dry steppe with *Stipa* and *Leymus*	0
Temp. grasslands & savannahs	28-07-2019-RU-ZK-4	Dry steppe with *Stipa* and *Leymus*	0
Temp. grasslands & savannahs	25-07-2019-RU-ZK-6	Periodically flooded meadow with *Carex*	4
Temp. grasslands & savannahs	29-07-2019-RU-ZK-15	Mountain steppe with *Leymus* and *Carex*	2
Temp. grasslands & savannahs	15-05-2022-RU-CB-75	Forb - feather meadow	2
Temp. grasslands & savannahs	15-05-2022-RU-CB-76	Feather grass steppe	0
Temp. grasslands & savannahs	15-05-2022-RU-CB-77	Feather grass steppe	0
Temp. grasslands & savannahs	15-05-2022-RU-CB-78	Cereal forb meadow with feather	3
Temp. grasslands & savannahs	18-06-2022-RU-KN-86	*Betula* - *Pinus* forest	4
Temp. grasslands & savannahs	20-06-2022-MN-Ulaanbaatar-92	Ruderal vegetation	2
Temp. grasslands & savannahs	21-06-2022-MN-Töv-93	*Larix* forest	6
Temp. grasslands & savannahs	22-06-2022-MN-Ulaanbaatar-94	*Picea* forest	2
Temp. grasslands & savannahs	22-06-2022-MN-Töv-95	*Cedrus* - *Picea* forest	0
Temp. grasslands & savannahs	21-06-2022-MN-Töv-96	*Betulafusca* forest	5
Temp. grasslands & savannahs	22-06-2022-MN-Ulaanbaatar-97	Flood plain	1
Temp. grasslands & savannahs	23-06-2022-MN-Ulaanbaatar-98	Under *Salix* near the river	2
Temp. grasslands & savannahs	23-06-2022-MN-Töv-99	*Salix* thicket	2
Temp. grasslands & savannahs	23-06-2022-MN-Töv-100	Meadow steppe	0
Temp. grasslands & savannahs	21-06-2022-MN-Ulaanbaatar-101	Steppe under grazing	0
Temp. grasslands & savannahs	23-06-2022-MN-Ulaanbaatar-102	*Ulmus* on the slope	0
Temp. grasslands & savannahs	15-07-2022-KZ-Shyngyrlau-103	Feather grass steppe	1
Temp. grasslands & savannahs	28-10-2022-UZ-Tashkent-119	Old agricultural field	1
Temp. grasslands & savannahs	01-11-2022-UZ-Samarqand-120	*Ulmus* forest	5
Temp. grasslands & savannahs	31-10-2022-UZ-Samarqand-121	Forest strip near vineyard	4
Temp. grasslands & savannahs	31-10-2022-UZ-Samarqand-122	*Populus*, *Acerpseudoplatanus*, *Quercus*, *Juglans* forest	0
Temp. grasslands & savannahs	23-10-2022-UZ-Tashkent-123	*Juglansregia* forest	0
Temp. grasslands & savannahs	28-10-2022-UZ-Sirdaryo-124	Cotton field canal	0
Temp. grasslands & savannahs	28-10-2022-UZ-Jizzax-127	Dry mountain steppe	0
Temp. grasslands & savannahs	25-10-2022-UZ-Fergana-128	Cane with *Populus* in canal near cotton field	0
Temp. grasslands & savannahs	26-10-2022-UZ-Namangan-129	Shrubland	1
Flooded grasslands	07-08-2019-RU-PK-7	Meadow / shrubland with *Carex*	2
Flooded grasslands	08-08-2019-RU-PK-10	Periodically flooded meadow with *Carex*	5
Flooded grasslands	05-08-2019-RU-AM-16	Meadow / deciduous forest with *Prunuspadus*	9
Flooded grasslands	05-08-2019-RU-AM-17	Mixed forest and shrubland	0
Flooded grasslands	20-07-2021-RU-HK-34	*Pinuskoraiensis* forest	5
Deserts & xeric shrublands	04-05-2022-UZ-Xorazm-67	*Larix* forest	1
Deserts & xeric shrublands	30-04-2022-UZ-Xorazm-68	Meadow near orchard	1
Deserts & xeric shrublands	30-04-2022-UZ-Xorazm-69	Cotton field canal	0
Deserts & xeric shrublands	03-05-2022-UZ-Karakalpakstan-70	Deciduous forest	0
Deserts & xeric shrublands	03-05-2022-UZ-Karakalpakstan-71	Ruderal vegetation	0
Deserts & xeric shrublands	02-05-2022-UZ-Karakalpakstan-72	Deciduous forest	0
Deserts & xeric shrublands	05-05-2022-UZ-Karakalpakstan-73	*Elaeagnusargentea* - *Turanga* thicket	0
Deserts & xeric shrublands	05-05-2022-UZ-Karakalpakstan-74	Dry canal bottom	0

**Table 2.** Average species richness per sampling site within a biome (m ± SE), total species richness and average abundance (indiv. per square metre ± SE) of enchytraeids in the studied biomes of the Asiatic part of the Northern Palaearctic. The numbers in brackets adjacent to specific biomes indicate the number of true replicates. Unidentified enchytraeids were excluded when counting the number of species. Specimens identified only at the genus level (Genus sp.) were included in the analysis as unique species, while juvenile specimens were only included in the counts if species from the same genus were absent at the site. The classification of biomes is given according to Olson et al. (2001).

**Biome**	**Average No of Species**	**Total No of Species**	**Average abundance**
Tundra (n = 13)	2.77 ± 0.47	16	7207 ± 3126
Boreal forests (n = 59)	2.31 ± 0.24	34	4296 ± 831
Temperate coniferous forests (n = 5)	3.4 ± 0.87	10	3789 ± 1044
Temperate broadleaf and mixed forests (n = 11)	3.64 ± 0.79	20	5725 ± 1767
Temperate grasslands, savannahs and shrublands (n = 30)	1.40 ± 0.29	19	2287 ± 516
Flooded grasslands and savannahs (n = 5)	4.00 ± 1.38	11	12288 ± 7644
Deserts and xeric shrublands (n = 8)	0.25 ± 0.16	2	384 ± 269
